# The relevance of aging-related changes in brain function to rehabilitation in aging-related disease

**DOI:** 10.3389/fnhum.2015.00307

**Published:** 2015-05-27

**Authors:** Bruce Crosson, Keith M. McGregor, Joe R. Nocera, Jonathan H. Drucker, Stella M. Tran, Andrew J. Butler

**Affiliations:** ^1^Department of Veterans Affairs Rehabilitation Research and Development Center of Excellence for Visual and Neurocognitive Rehabilitation (151R), Atlanta VA Medical CenterDecatur, GA, USA; ^2^Department of Neurology, Emory UniversityAtlanta, GA, USA; ^3^Department of Psychology, Georgia State UniversityAtlanta, GA, USA; ^4^School of Health and Rehabilitation Sciences, University of QueenslandBrisbane, Qld, Australia; ^5^Department of Psychology, Emory UniversityAtlanta, GA, USA; ^6^Department of Physical Therapy and School of Nursing and Health Professionals, Georgia State UniversityAtlanta, GA, USA

**Keywords:** rehabilitation, aging, stroke, neuroimaging, transcranial magnetic stimulation repetitive, transcranial direct current stimulation, aphasia, hemiplegia

## Abstract

The effects of aging on rehabilitation of aging-related diseases are rarely a design consideration in rehabilitation research. In this brief review we present strong coincidental evidence from these two fields suggesting that deficits in aging-related disease or injury are compounded by the interaction between aging-related brain changes and disease-related brain changes. Specifically, we hypothesize that some aphasia, motor, and neglect treatments using repetitive transcranial magnetic stimulation (rTMS) or transcranial direct current stimulation (tDCS) in stroke patients may address the aging side of this interaction. The importance of testing this hypothesis and addressing the larger aging by aging-related disease interaction is discussed. Underlying mechanisms in aging that most likely are relevant to rehabilitation of aging-related diseases also are covered.

In the past few years, new modalities have become available to manipulate activity in specific brain areas during rehabilitation. Primary among these intervention modalities are repetitive transcranial magnetic stimulation (rTMS) and transcranial direct current stimulation (tDCS). Recent studies indicate that these modalities can be used to enhance performance in motor or language tasks in stroke patients (e.g., Naeser et al., 2005, 2011; Baker et al., 2010; Barwood et al., 2011; Zimerman et al., 2012; Barros Galvão et al., 2014) or even in normal young or old participants (e.g., Kwon et al., 2013; Saucedo Marquez et al., 2013; Zimerman et al., 2013). Hence, these modalities have applicability to both language and motor rehabilitation. The canonical rationale for such interventions is that they are treating changes in brain systems that result from brain disease/injury, e.g., stroke.

In this brief review of aging and rehabilitation, we present evidence for a different viewpoint: in many instances, these interventions may be successful because they address the interaction between an aging-related disease state and normal aging-related changes in brain function *by impacting the aging component of the interaction*. We further suggest that attending to and understanding this interaction between disease state and aging will allow us to more rapidly and effectively develop new interventions using these and other intervention modalities. Since much of this work has involved remapping brain systems in stroke, we begin with a brief introduction to the noninvasive brain stimulation techniques used for this remapping (i.e., rTMS and tDCS), followed by a short discussion of stroke and aging. Subsequently, we present two examples in stroke rehabilitation where data suggest that intervention effects may be the result of addressing an aging-related phenomenon. Then, we will expand our discussion to other cognitive processes and aging-related diseases, and finally we discuss the implications for rehabilitation research in older persons.

## rTMS and tDCS

While rTMS and tDCS both can increase or decrease cortical excitability in the short run and help to remodel cortical systems in the long run, they do so by quite different mechanisms. While we discuss the underlying neural effects of these techniques later in this paper, a brief introduction is appropriate at this point. Transcranial magnetic stimulation (TMS) passes electrical current through a coil to create a brief magnetic field that penetrates the skull and induces an electrical current orthogonal to the magnetic pulse in underlying brain tissue. This current stimulates neurons in the target cortex, causing them to fire. In rTMS, stimulation is repeated in regular patterns. Low frequency rTMS reduces cortical excitability (Chen et al., 1997); 1 Hz is the most commonly used low frequency for rTMS. It is this reduction in excitability upon which the observations of the current discussion are based. High frequency rTMS increases cortical excitability (Wu et al., 2000); high frequency rTMS is usually performed at 10 Hz or above, but occasionally, frequencies as low as 5 Hz may be seen in the literature. Other types of rTMS (e.g., theta burst) are being used with increasing frequency in the literature. However, as they do not play a large role in the current discussion, they will not be discussed in detail here.

tDCS uses an anode and cathode from a direct current source. In anodal tDCS, the anode is placed above the target cortex, and the cathode is placed in a different location (e.g., a shoulder or a neutral location in the opposite hemisphere). For cathodal tDCS, the cathode is placed over the target cortex. At a cellular level, tDCS modifies the transmembrane potential by forcing the displacement of intracellular ions which cancel the generated intracellular field and thereby modify the spike firing probability (Bikson et al., 2004; Ruffini et al., 2013). With sufficient tDCS duration, synaptically driven aftereffects are induced (Bindman et al., 1964). Although anodal tDCS tends to increase cortical excitability and cathodal tDCS tends to decrease excitability, the effects on long-term remodeling are more complex (see discussion below). Similar to rTMS, different ways of delivering tDCS are being developed (e.g., using multiple anodes/cathodes to produce a more focal stimulation pattern), but these newer techniques will not play a large role in the current discussion.

## Aging and Stroke

During the past decade, studies of non-invasive brain stimulation (NIBS; i.e., rTMS, tDCS) to treat language and motor disorders after stroke have become increasingly prominent in the rehabilitation literature. Stroke is a disease primarily of older persons. The risk of stroke doubles for each decade of life between the ages of 55 and 85, and age is considered a risk factor for stroke (National Institute of Neruological Disorders and Stroke, 2014). These facts raise important questions about treating the consequences of stroke, particularly as it relates to language and motor deficits. Some questions are: How do aging-related changes in brain function affect language and motor systems? Do these changes interact with changes resulting from aging-related diseases, such as stroke? If so, what impact should this interaction have on the way in which we deliver rehabilitation? For example, it is well known that aging changes attention, memory, and efficiency and speed of cognitive processing and motor performance. It would be easy to see such aging-related changes as barriers to rehabilitation because they affect learning, making it more difficult to mount rehabilitation efforts. However, we assert that aging-related changes also may represent targets for rehabilitation. To the degree that aging-related changes combine with the effects of disease to create synergistic deficits, mitigating the aging-related component should provide one avenue to improving function. The following evidence suggests that there may be aging-related components to aphasia, to motor deficits, and to neglect after stroke and that this component may be amenable to intervention.

## Disease-Aging Interactions in Aphasia After Stroke

The use of NIBS in the treatment of post-stroke aphasia has shown promise. In one of the first studies in this area, Naeser et al. (2005) treated four patients with chronic (5–11 years post-stroke) nonfluent aphasia (mean age = 55.0 years) using low-frequency (1 Hz) rTMS targeting right pars triangularis in daily 20-min sessions for 10 days. As just noted, low frequency rTMS has been shown to decrease cortical excitability (Chen et al., 1997). They provided no further treatment to patients. All patients showed increased naming accuracy and decreased naming latency from baseline to immediately post-treatment, and improvement in picture naming continued for up to 8 months after the end of treatment. One problem that Naeser et al. (2005) noted with their initial study design was that no control group or control/placebo stimulation was used. Although spontaneous improvement was not likely to be great at 5–11 years post stroke, the lack of a control group left some doubt as to whether 1 Hz rTMS or some other variable (e.g., the social attention patients received during rTMS sessions) could have accounted for changes.

Barwood et al. (2011) used a two-parallel groups design to address the weakness in Naeser’s study. In one arm of the trial, six stroke survivors with chronic (2–10 years post stroke) nonfluent aphasia (mean age = 60.8 years) received 20 min of low frequency (1 Hz) rTMS to right pars triangularis daily for 10 days (like Naeser’s design). In the other arm, six chronic stroke survivors with nonfluent aphasia (mean age = 66.7 years) received sham rTMS on the same schedule. Again no further behavioral intervention was provided to either group. Significant group differences emerged in favor of the rTMS group from baseline to 1 week post-rTMS for naming accuracy, latency, repetition, and some narrative language measures. These findings suggest that Naeser et al. (2005) results were due to the effects of rTMS and not to some other factor.

Since these studies, Thiel et al. (2013) showed that low frequency (1 Hz, 20 min) rTMS aimed at right pars triangularis could improve the results of aphasia therapy when given right before it. For 10 days, 13 people recovering from aphasia (mean age = 69.8 years) received rTMS and 11 (mean age = 71.2 years) received sham rTMS before 45 min of language therapy (i.e., “deficit-specific aphasia therapy focused on the individual linguistic symptoms”). Naming and other language functions improved significantly more in the rTMS than the sham group. During H_2_^15^O PET scans of generating verbs compared to eyes-closed resting state, there were significantly different changes in laterality indices between the rTMS and sham control groups. The rTMS group showed a shift in cortical activity toward the left-hemisphere from pre- to post-treatment while the sham control group showed a smaller rightward shift in cortical activity.

Naeser et al. (2005), Barwood et al. (2011), and Thiel et al. (2013) all raised the possibility that right pars triangularis demonstrates reduced inhibition as a result of stroke and that this disinhibition impedes language functions in the chronic phase of aphasia. Therefore, they reasoned that use of 1 Hz rTMS to downregulate right pars triangularis activity would lead to greater inhibition of this right-hemisphere area, leading to therapeutic gain. Indeed, similar ideas regarding the origin of right-hemisphere activity in aphasia are prevalent in the neuroimaging literature. For example, Rosen et al. (2000) hypothesized that increased activity in the right inferior frontal gyrus during language tasks in aphasia was anomalous and related to a loss of “mechanisms that normally regulate the level of activation” in frontal regions homologous to left frontal language mechanisms. According to Rosen et al. (2000), such lost regulation could include active inhibition of the right inferior frontal gyrus, or right frontal activity in aphasia patients could reflect competitive interaction with its left frontal homolog.^1^ Similarly, Heiss and Thiel (2006) suggested that over-activation of right-hemisphere homologs to left-hemisphere language areas during overt language production in aphasia could “be interpreted as a result of decreased transcallosal inhibition due to damage” of left-lateralized language cortex.

To be clear, we do not dispute that activity in right pars triangularis in some cases appears to interfere with word finding. This proposition is the most straightforward interpretation of Naeser et al. (2005) and Barwood et al. (2011) data. Rather, we suggest that the origin of this right pars triangularis activity is not primarily a product of stroke affecting the left inferior frontal gyrus. Rather, we believe that increased right frontal activity in pars triangularis and neighboring areas is normal for aging. Indeed, as long ago as 1999, Warburton et al. (1999) noted that the right inferior frontal gyrus activity of aphasic patients during word generation was not outside the range of similar activity in age appropriate, neurologically normal controls. However, the importance of aging-related processes to this finding was not obvious since the authors did not also collect data on younger controls. We now turn to the importance of aging for right frontal activity during word generation.

**Figure 1:**
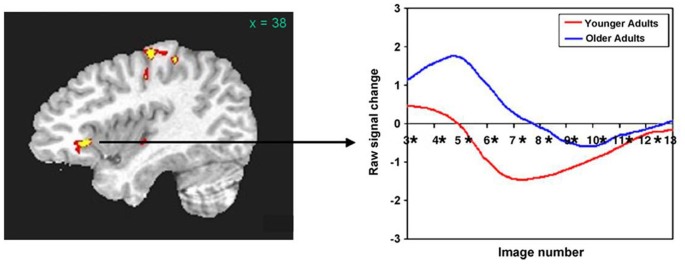
**(Left panel) The right pars triangularis activity difference between younger and older adults during picture naming (Wierenga et al., 2008) is half way up and just in front of the anterior ascending ramus of the Sylvian fissure. (Right panel)** Activity during naming for older subjects (blue line) in this region is positive (above baseline) compared to decreased (below baseline) for younger subjects (red line). The area of rTMS stimulation for aphasia treatment in Barwood et al. (2011) which is in precisely the same location as the activity difference between older and younger subjects in the Left panel. [Reprinted from Figure 3 of Wierenga et al. (2008), Copyright (2008), with permission from Elsevier].

Based upon recent data from our laboratory, we assert that the right frontal activity noted in older normal subjects may be primarily a product of aging. For example, Wierenga et al. (2008) compared activity of younger (mean age = 25.1 years) and older (mean age = 79.4 years) adults to naming black and white pictures vs. viewing pixelated (unrecognizable) versions of the same pictures. Right pars triangularis was among the areas showing differences between these groups. Figure 1 (left panel) shows that this activity difference is a few millimeters anterior to and about half way up the anterior ascending ramus of the right Sylvian fissure. Figure 1 (right panel) indicates that activity in the younger group was largely a negative blood oxygenation level dependent (BOLD) response, i.e., BOLD activity during picture naming was below the baseline during which subjects viewed the pixelated pictures. This pattern suggests a suppression of right pars triangularis during picture naming for younger subjects. However, the older subjects showed a positive BOLD response in this region during picture naming, suggesting that this region of cortex has become active during aging. The target of rTMS for Barwood et al. (2011) is in the same location as Wierenga’s activity difference between younger and older adults, i.e., a few millimeters forward of and about half way up the anterior ascending ramus of the right Sylvian fissure. Thus, loss of suppression of right pars triangularis during word production in the neurologically intact older brain appears to be a function of aging.

**Figure 2:**
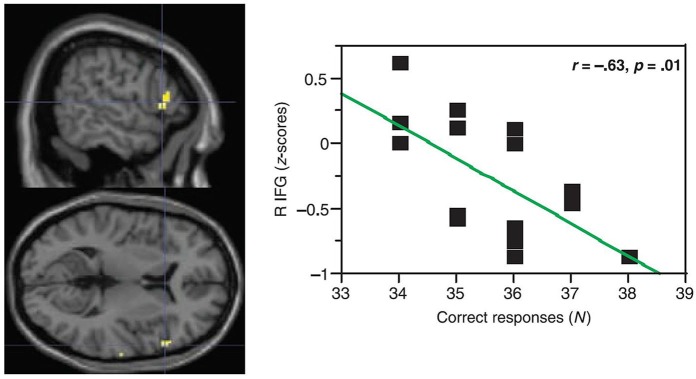
**In paced generation of category members (10 members for each of 4 categories) for older adults, Meinzer et al. (2009) showed a negative correlation between activity in right pars triangularis and accuracy of category member generation**. In other words, older participants with positive (above baseline) activity during category member generation showed worse performance (lower accuracy) than older participants with negative (below baseline) activity. Older participants with negative (below baseline activity) during category member generation showed higher accuracy. [Reprinted from Figure 3B of Meinzer et al. (2009), Copyright (2009), with permission from MIT Press].

One might question: What is the function of this activity increase in aging? The canonical interpretation for similar increases in activity is that it helps compensate for left-hemisphere mechanisms that are becoming less able as age advances (e.g., Cabeza, 2002). However, the data do not support this position. In simple picture naming, findings for Wierenga et al. (2008) were somewhat complicated. For high-performing older adults, right pars triangularis activity was positively correlated with accuracy, but for low performing older adults, right pars triangularis activity was negatively correlated with picture-naming accuracy. Findings were more straightforward for Meinzer et al. (2009) who compared paced generation of 10 members of four different categories to paced repetition of the word “rest”. They found activity differences between younger (mean age = 26.1 years) and older (mean age = 69.3 years) adults in pars triangularis and in the middle frontal gyrus. In both instances, increased right frontal positive BOLD activity was accompanied by decreased performance. Figure 2 shows this relationship in right pars triangularis. Meinzer and colleagues’ more monolithic relationship between word generation performance and right frontal activity [as opposed to the bimodal relationship in Wierenga et al. (2008) study] is probably related to the greater difficulty of generating multiple members of relatively discrete categories as opposed to naming pictures. Meinzer et al. (2012) replicated the finding that increased right frontal activity was related to poorer performance in category member generation for neurologically normal older (mean age = 69.2 years) adults. It should be noted that the highest performing older subjects in Meinzer and colleagues’ studies had activity below baseline representative of suppression, similar to younger adults in Wierenga et al. (2008) study.

These latter findings suggest that right frontal activity increases in neurologically normal older adults may interfere with word finding, albeit to a less dramatic extent than for stroke survivors with aphasia. Pursuant to these observations, we hypothesize that this interference with word finding, which is relatively subtle in normal aging, is exacerbated exponentially with compromise of the language eloquent left frontal cortex in acquired aphasia. If this is the case, it follows that, Naeser et al. (2005), Barwood et al. (2011), and Thiel et al. (2013) may actually have been treating an aging-related phenomenon that was compromising word retrieval in a synergistic fashion with the damage of language-eloquent cortex as a result of stroke. This insight raises the question of whether other aging-related phenomena may interact with aphasia and whether new treatment approaches can be used to mitigate the synergistic deficits. For example, Meinzer et al. (2012) also found increased activity in right posterior perisylvian cortex for older compared to younger adults, and such increases in activity were associated with decreased category member generation accuracy for older adults. Could low-frequency rTMS of the right posterior perisylvian regions downregulate activity in this region and improve language functions for patients with fluent aphasias after left posterior perisylvian lesions? Alzheimer’s disease (AD) might be another aging-related disease where this strategy might be used. In patients with mild AD or amnestic mild cognitive impairment (aMCI), Moffett et al. (2012) showed that increased activity in right posterior perisylvian cortex was associated with poorer performance on semantic tasks. While high frequency rTMS of left dorsolateral prefrontal cortex has been used for language deficits in AD (e.g., Cotelli et al., 2006, 2008, 2011), we know of no studies using low frequency rTMS in this region of the right hemisphere for AD.

One final point is that reduced inhibition in aging is likely not to be confined to the right hemisphere, or to language systems for that matter. Indeed, low frequency rTMS has been applied to the left as well as the right hemisphere in patients with aphasia, either alone (Kakuda et al., 2010) or prior to language therapy (Abo et al., 2012). For individual patients in these studies, the hemisphere to which rTMS was applied was the hemisphere showing less activity than the other during functional imaging of a repetition task. For Abo et al. (2012) the inferior frontal gyrus was stimulated in nonfluent patients, and the superior temporal gyrus was stimulated for fluent patients. While these studies report positive findings, one must be cautious in interpreting results since a sham control group was not used. Nonetheless, the possibility that low frequency rTMS of left-hemisphere structures can work in some circumstances is intriguing and suggests that loss of inhibitory mechanisms may be a more general aging-related phenomenon interacting with stroke. Indeed, it can be found in the motor system, a topic to which we now turn.

## Disease-Aging Interactions in Motor Cortex After Stroke

Hemiplegia and hemiparesis also are common after stroke. Over the past few years, studies using rTMS (e.g., Etoh et al., 2013; Kwon et al., 2013; Tretriluxana et al., 2013; Barros Galvão et al., 2014) and tDCS (e.g., Wu et al., 2013; or see Butler et al., 2013) to mitigate motor impairment after stroke have become abundant. One way in which rTMS and tDCS have been used is to apply low frequency rTMS or cathodal tDCS over the hand-motor cortex of the undamaged hemisphere (i.e., the motor cortex ipsilateral to the deficit). This form of rTMS has been used successfully alone to improve motor function in the affected upper extremity (e.g., Grefkes et al., 2010; Kondo et al., 2013; Tretriluxana et al., 2013) and also to enhance the effects of upper extremity therapies (e.g., Conforto et al., 2012; Etoh et al., 2013; Barros Galvão et al., 2014). Again, low frequency rTMS downregulates the excitability of the target cortex and cathodal tDCS is thought to do the same (Lang et al., 2004; but see Simis et al., 2013). It should be noted that there occasionally have been failed trials of rTMS used in this fashion (e.g., Seniów et al., 2012). High frequency (excitatory) rTMS of perilesional cortex in the affected hemisphere (Emara et al., 2010; Khedr et al., 2010) and combined low frequency rTMS of the unaffected hemisphere and high frequency rTMS or intermittent theta burst rTMS (which also increases cortical excitability) of the affected hemisphere (e.g., Sung et al., 2013; Yamada et al., 2013) also have yielded results. The putative mechanism for the successful trials has been reviewed by Corti et al. (2012). In short, decreased cortical excitation in perilesional cortex is thought to result in decreased transcallosal excitation of inhibition in the unaffected hemisphere, leading to over-excitation in the unaffected motor cortices. This over-excitation in the unaffected hemisphere, in turn, excites greater inhibition in the affected motor cortices via the corpus callosum, decreasing their ability to initiate motor activity. Hence, either by increasing excitability in affected motor cortices or by decreasing excitability in unaffected motor cortices (or both), the balance between inhibitory and excitatory activity in perilesional cortex can be restored, at least partially.

**Figure 3:**
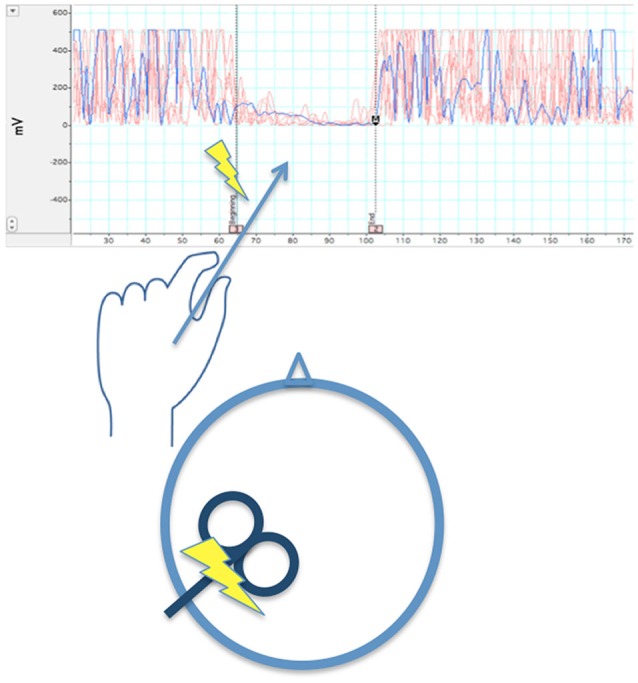
**When left M1 is stimulated by a single TMS pulse during isometric contraction of the left first dorsal interosseous muscle, after a delay >40 ms (~65 ms in the illustration), there is a temporary reduction of EMG activity in that muscle**. The length the reduction in EMG activity is averaged across several trials to obtain a stable iSP.

It is worth considering how well data regarding intra- and inter-hemispheric inhibition after M1 strokes support this hypothesis. Below we show difficulties regarding this theory as demonstrated by the literature. Before reviewing this research, it is important to note methodological considerations in the way intra- and inter-hemispheric inhibition are measured. (1) The first consideration involves the use of single- vs. paired-pulse TMS. In single-pulse TMS (Figure 3), the pulse is delivered to M1 during isometric contraction of the target muscle. Silent periods (SP; i.e., reduction of EMG activity) can be noted in either muscles ipsilateral to the stimulated M1 (ipsilateral silent period: iSP) or contralateral to the stimulated M1 (contralateral or cortical silent period: cSP).

**Figure 4:**
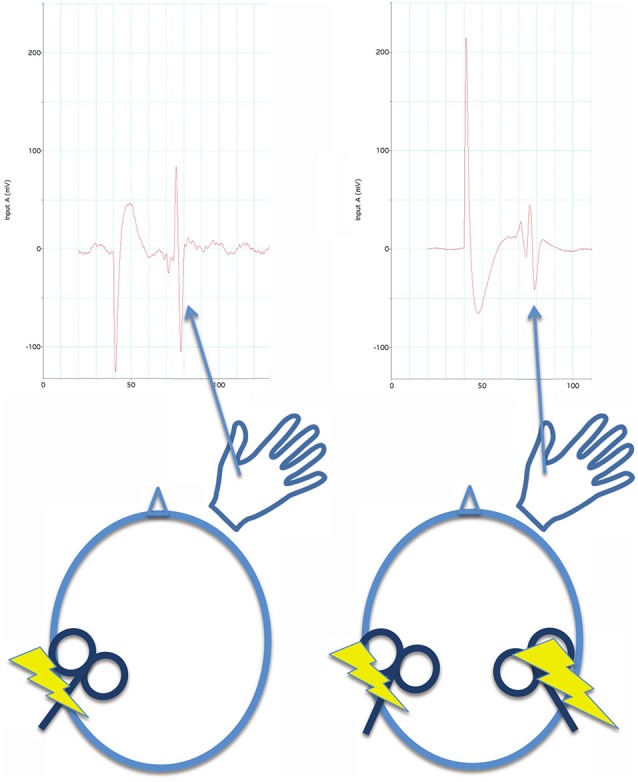
**For paired pulse inhibition, a reference evoked EMG potential is elicited from the first dorsal interosseous muscle by a single pulse of TMS to the opposite M1 for reference (left side of figure)**. Then, a conditioning stimulus (single TMS pulse) is applied to the contralateral M1 before (40 ms before in this illustration) the test stimulus (also a single TMS pulse). At this interval, inhibition is measured by a reduction in the evoked EMG potential to the test stimulus (right side of figure) in comparison to the reference evoked potential. Both reference and paired-pulse evoked potentials are averaged across several trials to obtain stable measures.

iSP measures transcallosal (interhemispheric) inhibition, cSP measures intrahemispheric inhibition. In paired pulse TMS (Figure 4), a pulse or conditioning stimulus (CS) is applied shortly before a test stimulus (TS). Applying the CS and TS to the same M1 can be used to measure intrahemispheric inhibition, and applying the CS and TS to opposite M1s can be used to measure interhemsipheric inhibition. The effect of inhibition aroused by the CS is measured by reduction of the evoked response in the TS. (2) Generally, inhibition measured at short inter-pulse intervals (a few milliseconds) in paired-pulse paradigms is mediated by GABA_A_ receptors, which are ionotropic. Inhibition measured by paired-pulse paradigms at long intervals (tens of milliseconds) and silent period after a single pulse are mediated by GABA_B_ receptors, which are metabatropic (see discussion below). (3) The inhibition can be measured while the muscle is at rest, during an isometric contraction, or prior to movement of the target muscle.

Regarding the model for rTMS effects as discussed by Corti et al. (2012), some elements have been supported, while others have not. The idea that there is decreased excitation in perilesional cortex after stroke is not well-supported by animal studies showing reduced GABA_A_ receptors and increased excitability of cortical neurons close to experimentally induced ischemia (e.g., Schiene et al., 1996), though it is difficult to disentangle whether increased excitability relates to interhemispheric connections or intrahemispheric connections. The idea that interhemispheric inhibition from the affected to the unaffected hemisphere is reduced has been supported by human paired-pulse studies. In stroke patients in the subacute phase of recovery (i.e., in the first month post-stroke), Bütefisch et al. (2008) found decreased interhemispheric inhibition from the affected to the unaffected hemisphere in stroke survivors (mean age = 56.75 years) relative to age-matched controls (mean age = 50.20 years) at 10 ms, but not at shorter inter-pulse intervals. Boroojerdi et al. (1996) found decreased interhemispheric inhibition from the affected to the unaffected hemisphere at 7, 15, and 30 ms after the CS in patients with lesions affecting both cortical (mean age = 62.20 years) and subcortical (mean age = 59.41 years) structures. Nonetheless, the idea of increased interhemispheric inhibition of the unaffected on the affected hemisphere, the key element of the theory, has not been supported by at least one paired-pulse study at rest. Specifically, Bütefisch et al. (2008) did not find significant differences with age-matched controls in interhemispheric inhibition from the unaffected to the affected hemisphere. It should be noted, however, that this study was not designed to detect an interaction between aging and stroke. That inquiry would have required the addition of a young control group. Hence, it is possible that greater inhibition of the affected by the unaffected hemisphere could occur as a function of aging, and that it is this function which low-frequency rTMS of the unaffected M1 addresses. We shall discuss data supporting this contention shortly.

It is worth noting that a single session training forward reaching in the affected arm in chronic (mean = 7 years post) stroke survivors (mean age = 54.9 years) results in improved reaching, reduced amplitude of iSP, and reduced duration of cSP in the affected triceps (Harris-Love et al., 2011), which generally is consistent with the fact that rTMS of the unaffected M1 improves movement of the affected hand. In a study of stroke survivors (mean age = 62 years) selected for abnormally long cSPs, Classen et al. (1997) showed that motor improvements over time in these patients were accompanied by reduction of cSP to normal levels (Experiments quantifying inhibition used the first dorsal interosseus muscle to measure inhibition, unless otherwise specified).

The above TMS studies measured inhibition in a resting state or during isometric contraction. Murase et al. (2004) used paired-pulse TMS (20–40 ms inter-pulse interval) to measure inhibition at the first dorsal interosseus muscle of the affected hand of stroke patients (mean age = 65 years) after a “go” signal to move their affected index finger, but before the first EMG changes heralding movement. Close to the “go” signal, they found interhemispheric inhibition of the unaffected on the affected hemisphere in stroke patients and age-matched controls (mean age = 62 years). As measurements approached the time of movement, the interhemispheric effects in age-matched controls changed from inhibition to facilitation, whereas in the affected hand of stroke patients, the response approached a neutral point between inhibition and facilitation. In light of studies discussed above, findings of this study suggest that movement of the impaired hand of people recovering from stroke may induce processes that rest and isometric contractions do not, and an argument can be made that this paradigm is more appropriate to determine the effects of interhemispheric inhibition during movement. Nonetheless, this experiment was not designed to detect how aging affects inhibition during movement. It is impossible to determine such aging-related effects without a young control group. Clearly, more research is needed to unravel what inhibitory effects are due to lesion alone and which may be due to the interaction of stroke and aging.

Below, we turn to data establishing aging-related effects on interhemispheric inhibition between primary motor cortices. Since most stroke survivors are older (i.e., greater than 40 years of age), these data indicate a need to incorporate age into models of interhemispheric inhibition after stroke. We do not question that the activity in the unaffected hemisphere during motor responses of the affected hand may be detrimental to movement initiation, accuracy, and speed. However, we urge reconsideration of the concept that over-activity in the unaffected hemisphere is exclusively, or even primarily due to mechanisms emerging from stroke.

Specifically, recent research in aging has shown that activity in motor cortex (M1) ipsilateral to a moving hand is suppressed in neurologically normal younger adults but increased in neurologically normal older people. Using functional magnetic resonance imaging (fMRI), McGregor et al. (2009) found that BOLD activity in cortex ipsilateral to moving fingers was negative (below a baseline in which the hands were still) for six neurologically normal younger volunteers (mean age = 22 years), but positive (increased above baseline) in five of six neurologically normal older adults (mean age = 71 years). It did not matter whether the movements were internally or externally guided. A curious finding from this study was that the one older adult who engaged in extremely high levels of aerobic activity was the older subject with a response similar to that of younger persons, i.e., showed a negative BOLD response in ipsilateral M1 when the right hand was moving.

**Figure 5:**
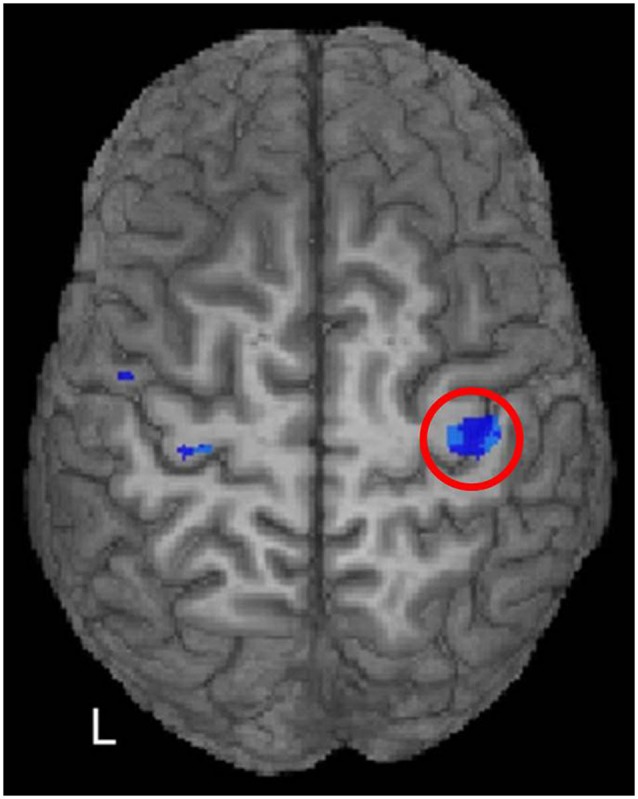
**McGregor et al. (2011) correlated fMRI activity during right index finger tapping with the iSP from left M1 stimulation in younger and older adults**. A region of significant negative correlation (*p* < 0.05, FDR corrected) of right M1 activity with iSP is shown in blue hues within the red circle. In other words, positive (above baseline) fMRI activity was associated with shorter iSPs than negative (below baseline) activity. [Reprinted from Figure 4 of McGregor et al. (2011), Copyright (2011), with permission from Elsevier].

McGregor et al. (2011) wondered whether or not this phenomenon (negative BOLD in M1 ipsilateral to moving fingers for younger subjects, but positive BOLD under the same conditions for older subjects) was related to inhibitory mechanisms as measured with single-pulse TMS and whether a history of exercise impacted BOLD responses and inhibition evoked by TMS. They recruited groups of young adults (average age = 24.30 years), sedentary older adults (average age = 70.64 years; <45 min/week of moderate to strenuous exercise), and older adults with a history of regular moderate to strenuous exercise (average age = 68.20 years; >30 min of moderate to strenuous exercise at least 3 times/week). In addition to fMRI during finger movement, they measured the iSP from the first dorsal interosseous muscle of the left hand using single-pulse TMS of left M1. In this paradigm, participants squeezed a force transducer between their left thumb and forefinger at 40–50% of maximum voluntary contraction during single-pulse TMS of left M1. As mentioned above, the iSP is a period of decreased EMG activity beginning within a few tens of milliseconds after the TMS pulse to ipsilateral M1 and is a measure of inhibition induced by cross-callosal mechanisms. The iSP is considered to be related to GABAergic mechanisms (GABA is the most abundant inhibitory neurotransmitter in the nervous system), since iSP and related cortical inhibitory mechanisms can be strengthened by GABA_B_ receptor agonists (e.g., Siebner et al., 1998; Irlbacher et al., 2007). In the entire sample of younger and older subjects (as well as in the older individuals alone), McGregor et al. (2011, 2013) found that positive BOLD responses in right M1 during finger movements were associated with shorter iSPs in the left hand during left M1 TMS, and negative BOLD responses were associated with longer iSPs (Figure 5). This negative correlation between amplitude of BOLD response and length of iSP established a link between negative BOLD responses in M1 and transcallosal inhibitory mechanisms. McGregor et al. (2009) also found that loss of negative BOLD and shortening of the iSP both were mitigated in active compared to sedentary older adults, indicating that a history of moderate to strenuous exercise was protective against the effects of aging. McGregor and colleagues’ findings regarding shortening of the iSP with aging, its relationship to motor performance, and change in BOLD signal with aging have been confirmed by studies from other laboratories (e.g., Riecker et al., 2006; Davidson and Tremblay, 2013).

Subsequently, these investigators extended their findings in older subjects to persons at middle age, i.e., 40–60 years of age (mean = 52.1 years; McGregor et al., 2013). Previous findings in older adults were replicated; i.e., sedentary adults at middle age showed greater BOLD responses and shorter iSPs than either fit adults at middle age or younger adults, and iSP for the left hand correlated with negative BOLD in right M1 during movement of fingers of the right hand. In addition to this replication, several new findings were of interest. None of the differences between sedentary and fit adults at middle age were found between fit and sedentary young adults, indicating that the differences between fit and sedentary adults at middle age were a function of aging. In adults at middle age, length of iSP showed a positive correlation with VO_2max_ (maximum amount of oxygen consumed during aerobic exercise, a measure of fitness) and right M1 BOLD responses during movement of the right hand showed a negative correlation with VO_2max_. These correlations were not present for younger adults (mean age = 21 years; McGregor et al., 2012). Finally, fit adults at middle age showed greater manual dexterity than sedentary adults at middle age, and iSP was correlated with dexterity in these adults.

These data clearly indicate that reduction of M1 inhibition during movement of the ipsilateral hand is a function of aging. Indeed, these findings are not limited to adults above the age of 60; they can be found as early as 40 years of age. Hence, as in the language example given above, loss of inhibition of ipsilateral M1 during unilateral hand movements is not simply a function of loss of neuronal control mechanisms following stroke, but is a function of aging. However, cardiovascular fitness can mitigate these changes. Changes in physiological responses (iSP) are correlated with changes in behavior (dexterity), suggesting that the physiological changes have consequences in terms of motor performance. Given the fact that decreasing cortical excitability in the intact M1 of stroke patients with low-frequency rTMS or cathodal tDCS improves motor performance and/or response to therapy, we hypothesize that the loss of inhibition of M1 ipsilateral to a moving hand due to aging has a synergistic effect with stroke, increasing motor disability in the affected hand beyond what stroke-related damage does. Further, we hypothesize that reducing cortical excitability in contralesional M1 as an intervention (with rTMS or tDCS) is addressing, at least in part, the aging component of an aging by disease interaction in stroke.

## Underlying Mechanisms of Lost Cortical Suppression in Aging

Understanding mechanisms underlying loss of cortical suppression in aging is critical for developing interventions to address it. The most relevant candidate neurotransmitter system to explain the differences shown between age groups is the GABAergic system. GABA is the dominant inhibitory neurotransmitter system within the central nervous system and has powerful effects on cortical activity (Sohal et al., 2009). Recent literature implicates GABAergic mechanisms in negative (below baseline) BOLD responses (Northoff et al., 2007; Stagg et al., 2011). Further, pharmacologic challenges implicate specific GABA receptors in short and long inhibitory responses to TMS. Given other evidence that GABAergic mechanisms change with aging, the evidence is mounting that GABAergic mechanisms play a major role in aging-related changes in suppression of cortical responses and other inhibitory mechanisms. We review some of this evidence in the paragraphs that follow and then relate this evidence to the NIBS techniques we have been discussing.

In fMRI investigations, negative (below baseline) BOLD responses are indicative of cortical suppression (Shmuel et al., 2006; Klingner et al., 2012), and research into the relationship between cortical suppression in M1 and iSPs provides one clue as to these mechanisms. As noted above, McGregor et al. (2011, 2013) found that in aging, larger negative BOLD responses in M1 ipsilateral to a moving hand are correlated with longer inhibition (i.e., iSPs). This link between neuroimaging and neurophysiological data sources provides strong evidence that sedentary aging confers some alteration of inhibitory cortical function. However, these findings are limited without insight into the underlying mechanism by which this loss of inhibition may manifest as a result of the aging process. To begin to address this, in the following paragraphs, we: (1) will link these phenomena to GABAergic inhibition; (2) will discuss additional evidence of changes in GABAergic activity in aging; and (3) will show that inferences regarding GABAergic changes in aging extend beyond M1 and the motor system. Our aim is to provide enough evidence to strongly implicate GABAergic mechanisms as playing a major role in loss of cortical suppression.

Within the motor cortex, one of the most studied influences of inhibition is that of interhemispheric connectivity between the left and right primary motor areas. Though debated for some time, direct connections between the primary motor areas exist via transcallosal connectivity within the body of the corpus callosum (Wahl et al., 2007; Fabri et al., 2014). In younger adults, the communication between the left and right motor areas is inhibitory (Meyer et al., 1995; Boroojerdi et al., 1996; Tzourio-Mazoyer et al., 2015). Because callosal fibers consist largely of excitatory fibers, this transcallosal inhibition is thought to take place by termination of transcallosal fibers on GABA-mediated inhibitory interneurons (e.g., Swadlow, 2000).

GABAergic transmission is differentiated by receptor subtype of the neurotransmitter. The receptor subtypes are classified as GABA_A_ and GABA_B_. GABA_A_ receptors, which have multiple intrinsic subtypes, are ligand gated, i.e., ionotropic in nature. As such, they have fast acting effects when bound. GABA_A_ receptor activity is implicated in shorter latency inhibitory responses, such as TMS protocols involving short intracortical inhibition (SICI; e.g., Di Lazzaro et al., 2005, 2006; Premoli et al., 2014). SICI refers to a paradigm where M1 within a hemisphere is stimulated by two TMS pulses, one rapidly following the other at an interval of 1–5 ms. The first (conditioning) pulse creates a decrease in amplitude of the motor evoked potential (MEP) associated with the second (test) pulse, indicating inhibitory activity as a result of the conditioning pulse at the short interval between pulses. SICI performed during a resting state has been shown to be decreased in older adults (Peinemann et al., 2001; Marneweck et al., 2011; Heise et al., 2013, 2014; Fujiyama et al., 2014; Papegaaij et al., 2014).

Alternately, GABA_B_ receptors are relatively slow-acting metabotrobic (second messenger) receptors. GABA_B_ receptors have been implicated as a primary mechanism in the SP described above because the SP is prolonged in a dose-dependent manner by agents that facilitate GABA_B_ transmission (Thompson and Gähwiler, 1992; Siebner et al., 1998; Werhahn et al., 1999; Irlbacher et al., 2007). Further, the time course of the iSP (and the contralateral SP) is comparable to the inhibitory postsynaptic potential (IPSP) generated by the GABA_B_ receptor (Roick et al., 1993). As noted elsewhere in this review, McGregor et al. (2011, 2013) have found not only that the iSP is shortened in aging, but also that shorter iSPs in aging are associated with a change in BOLD responses from negative (below baseline) to positive (above baseline) in M1 ipsilateral to a moving hand. Hence, GABA_B_ receptors may be responsible for loss of a different type of inhibition and, most likely, in the corresponding loss of cortical activity suppression. Hence, loss of both GABA_A_-related and GABA_B_-related inhibition affect M1 in aging.

Further, GABAergic association with negative BOLD appears to extend beyond the motor system. In particular, both Northoff et al. (2007) and Hu et al. (2013) have shown that larger task-related negative BOLD responses in the anterior cingulate cortex and the retrosplenial portion of the default network (i.e., a network of brain regions that are active when the individual is not focused on generating responses to internal or external stimuli), respectively, are associated with the presence of greater GABA concentrations, as measured by resting nuclear magnetic resonance (NMR) spectroscopy. In our own lab, we have shown an aging-related loss of negative BOLD activity in the posterior cingulate/precuneus region, as well as the lateral portions of the default network (Meinzer et al., 2012). While this loss of negative BOLD in posterior cingulate cortex/precuneus may be related to amyloid deposits (Sperling et al., 2009) as well as loss of GABAergic activity, we have shown in aging that loss of negative BOLD activity in lateral regions of the default network is associated with shortened iSP’s, linking this loss of negative BOLD response to a GABA_B_-ergic phenomenon (Zlatar et al., 2013). These findings raise the possibility that changes in GABAergic mechanisms during aging extend beyond M1. However, more research in this area is needed.

Evidence of reduced GABAergic activity in aging is not limited to TMS and fMRI studies. Studies in animals have shown a loss of GABA projection neurons and interneurons in aging (Madhusudan et al., 2009; Schmidt et al., 2010; McQuail et al., 2012; Stanley et al., 2012). Further, the fact that GABA and GABA_A_ agonists can normalize activity in the visual cortex of older primates (Leventhal et al., 2003) suggests not only that GABAergic mechanisms have cross-species relevance but also that a lack of availability of GABA may play a role in aging. Yet, it is our impression that the effects of GABAergic mechanisms on aging-related cognition and behavior is an under-studied area, and that the studies mentioned above are only a start. Further study of these mechanisms, particularly in humans, would be welcome. NMR spectroscopy and GABA modulators are tools that would be useful in this endeavor.

Finally, it is possible that GABAergic mechanisms could be related to decreased neural plasticity in aging. Hayama et al. (2013) have shown that GABA promotes shrinkage and even elimination of spines (excitatory synaptic contacts) on dendrites of pyramidal neurons in the hippocampus (CA1) during long-term depression. Glutamatergic stimulation in a long-term potentiation protocol induces spine enlargement, out-competing GABAergic/long-term depression mechanisms when both are simultaneously active. These processes may be responsible for selective synaptogenesis during learning, and to the degree that they can be applied to learning during rehabilitation, could promote the neural plasticity underlying positive outcomes. If this is the case, then loss of GABAergic substrates in aging would make learning more difficult. For example, Moore et al. (2012) performed a stroke recovery analog study on young and middle-aged macaques. Older macaques took nearly twice as long to recover a learned fine motor skill (130–150 days) as young macaques (65–80 days). Since hippocampal and motor systems are not tightly linked, the effects of GABAergic activity on selective synaptogenesis and on learning in rehabilitation require further study.

In the discussion above, we have established that GABAergic mechanisms can be related to reduced short and long inhibitory responses evoked by TMS in aging. It is appropriate to point out that there are many other questions that can be addressed about the nature of the mechanisms: What is the nature of aging-related changes in intra- vs. inter-hemispheric inhibitory responses? What is the impact of GABAergic changes on sensory processing, various motor responses, and cognitive activity? The literature on these and related questions is sometimes sparse and frequently difficult to interpret because of the variety of paradigms. Hence, it is beyond the scope of this paper and must be left as the topic of another review. For our purposes, it is adequate to establish that GABAergic mechanisms are a major player in the phenomena we are discussing.

## GABAergic Mechanisms in NIBS

The GABAergic mechanisms discussed above play an important role in the NIBS techniques that we have been discussing. The reader will recall that we have hypothesized the effects of low frequency rTMS in language and motor systems after stroke are due to the aging component of an aging × disease interaction. Hence, it is important to shed some light on the behavior of GABAergic mechanisms in NIBS. Toward that purpose, we note that rTMS and tDCS sometimes have similar effects, though through distinct mechanisms of action.

TMS induces an electrical current that is sufficient to depolarize cells in the target area (Nitsche et al., 2008; Clark et al., 2011). Kozyrev et al. (2014) measured the effects of TMS at various frequencies on cortical membrane potentials *in vivo* in anesthetized cats. After a single pulse, brief focal activation was immediately followed by more widespread suppression. At 10 Hz, activity within this “basin of suppression” increased beyond the initial baseline to suprathreshold levels. As a result of the 10 Hz rTMS, spontaneous activity was increased, and sensory stimulation led to enhanced long-term potentiation of evoked activity. Based on these findings, the authors inferred the involvement of GABAergic interneuron networks in the following fashion: low frequency rTMS or single-pulse TMS engaged these networks, leading to overall cortical suppression, whereas high-frequency rTMS truncated the inhibitory response, disrupting the GABAergic networks and leading to overall cortical excitation.

Whereas TMS generates action potentials directly by phasically depolarizing neurons, tDCS is thought to act on firing rates indirectly by tonically increasing or decreasing membrane potentials in the target area (Nitsche et al., 2008; Clark et al., 2011). Magnetic resonance spectroscopy (MRS) following tDCS administration implicates a direct effect on glutamatergic transmission and an indirect effect on GABAergic transmission. One study found increased glutamate levels 30 min after anodal stimulation at 2.0 mA (Clark et al., 2011). Conversely, another study found reduced glutamate levels following cathodal stimulation. The same study found reduced GABA levels following both anodal and cathodal stimulation (Stagg et al., 2009). However, the lasting effects of tDCS may be mediated by transmission at the voltage-dependent NMDA receptor. The NMDA agonist C-cycloserine enhanced the effects of tDCS (Nitsche et al., 2004), whereas the NMDA antagonist dextromethorphan suppressed the effects of tDCS (Liebetanz et al., 2002). Nonetheless, in the same study, it was found that blocking sodium channels (i.e., both NMDA and AMPA subtypes) eliminated the effects of anodal, but not cathodal, tDCS. The complexity of the glutamatergic and GABAergic interaction could explain some of the seemingly contradictory findings for tDCS in the stroke literature (e.g., see the discussion below of similar effects for anodal and cathodal tDCS to left frontal cortex in aphasia).

## Other Potential Examples of Disease-Aging Interactions

To this point, we have concentrated on the relationship between aging-related cortical inhibitory changes and rehabilitation of language and motor deficits after stroke. However, there are other examples of interactions between aging-related and disease-related processes that can impact rehabilitation. Such examples can be found for other deficits in stroke and for other aging-related diseases. We give three examples below, starting with neglect in stroke, moving on to AD, and ending with a discussion of the potential effects of amyloid burden in stroke patients who were cognitively normal prior to stroke.

Right-hemisphere stroke can result in spatial neglect, a subtype of neglect characterized by a failure to attend, orient, and respond to the space contralateral to the lesioned hemisphere in the absence of basic motor and sensory deficits (Heilman et al., 2011). The parietal lobe is often involved in this subtype of neglect. Two common ways to measure the degree of visuospatial neglect are by strong rightward perceptual bias in the line bisection task and Landmark task (Milner et al., 1993; Adair and Barrett, 2008). Whereas right-sided neglect occurs rarely as result of structural damage to left perisylvian or subcortical regions, lesions in the right hemisphere are overwhelmingly more likely to cause both acute and enduring left-sided neglect, indicating that the right hemisphere maintains representations of both left and right hemispace, while the left hemisphere is more sensitive to the right hemispace (Heilman et al., 2011). In this sense, the right hemisphere can be thought of as dominant for visuospatial attention.

Similar to the reciprocal interhemispheric inhibition in language and motor networks, bilateral areas implicated in visuospatial neglect also demonstrate hemispheric rivalry in which activity in the right hemisphere may suppress homologous activation in the opposite hemisphere (Kinsbourne, 1977; Payne and Rushmore, 2004; Koch et al., 2011). Neglect is purportedly a result of depressed activity of the affected hemisphere (usually the right) in addition to disinhibition of the unaffected hemisphere. On the basis of this account, rTMS and tDCS have been applied to counteract the imbalance of activation by inhibiting the unaffected hemisphere (Oliveri et al., 2001; Corbetta et al., 2005; Sparing et al., 2009; Brem et al., 2014). Indeed, inhibitory cathodal tDCS applied to the intact left posterior parietal cortex and excitatory anodal tDCS applied to the affected right posterior parietal cortex reduced visuospatial neglect symptoms in a line bisection task though not in the neglect subtest of the Test Battery of Attentional Performance (Sparing et al., 2009). Using rTMS to inhibit the unaffected hemisphere in a region posterior to the intraparietal sulcus, Oliveri et al. (2001) showed that unilaterally brain damaged patients with contralesional neglect demonstrated improvement in the Landmark task. Their findings are corroborated by more recent studies applying the same intervention (for a review see Cazzoli et al., 2010).

Recent evidence suggests that visuospatial processing is subject to age-related change as demonstrated by changes in pseudoneglect (Jewell and McCourt, 2000; Failla et al., 2003; Pierce et al., 2003; Barrett and Craver-Lemley, 2008; Schmitz and Peigneux, 2011; Benwell et al., 2014). In normal young adults, pseudoneglect is characterized by a leftward perceptual bias (Bowers and Heilman, 1980). Schmitz and Peigneux (2011) found that with increasing age, the leftward perceptual bias demonstrated by young adults becomes suppressed and nearly reversed, such that older adults exhibit a pattern of rightward perceptual bias similar to but not as severe as that of patients with visuospatial neglect. Hence, older adults may be showing a decrease of inhibition of the left by the right posterior parietal lobule, similar to what we described above for motor and language functions. This analysis raises the possibility that the positive findings for cathodal tDCS and low-frequency rTMS of the left posterior parietal lobe for neglect are due to treatment of aging-related brain changes compounded by stroke-related brain changes. Indeed, the likelihood and severity of neglect resulting from right hemisphere stroke increases in older adults relative to younger adults, which may reflect the aging and aging-related disease interaction (Gottesman et al., 2008).

Besides stroke, other examples of aging-related disease that have shown response to rehabilitation include are: Parkinson’s disease, mild cognitive impairment, and even AD. Although there has been some tendency to regard degenerative diseases as poor targets for rehabilitation, there is promising recent research to indicate rehabilitation can induce lasting behavioral and brain changes in aMCI (e.g., Hampstead et al., 2008, 2011, 2012), Parkinson’s disease (e.g., Hackney and Earhart, 2008, 2010; McKee and Hackney, 2013), and even AD (e.g., Rothi et al., 2009). While the relationship of such interventions to underlying disease progression is uncertain, such treatments have the potential to reduce impairment and extend cognitive and motor capacity. Given the current momentum of this research, it is appropriate to consider how it might benefit from addressing the interaction between aging and aging-related disease.

For example, almost all people diagnosed with the degenerative aging-related disorder of AD will eventually show word-finding problems and language disturbances. In neurologically normal older adults, Meinzer et al. (2012) found a negative correlation between BOLD response in the right anterior supramarginal gyrus during category member generation and accuracy of performance in that task. Essentially, older adults who showed negative BOLD responses had greater accuracy than those who showed positive BOLD responses. Further, similar correlations were shown in right inferior parietal and superior temporal cortex during generation of words beginning with a given letter. Preliminary data from our laboratory have shown that this phenomenon in right posterior perisylvian cortex extends to aMCI and early AD. Moffett et al. (2012) showed that activity in right posterior perisylvian cortex during picture naming was negatively correlated with accuracy in that task in a sample of older adults that included the continuum from normal cognitive aging to aMCI to AD, indicating that Meinzer et al. (2012) findings extend all the way into AD. There were no such findings for left perisylvian regions. Hence, this early decline of naming appears to be related more to over-activity in right posterior perisylvian regions than to changes in left-hemisphere language-eloquent cortex, and it is likely, in our opinion, that this right-hemisphere phenomenon exacerbates later changes in language functions as compromise affects language eloquent cortex in AD. Interestingly, Rothi et al. (2009) demonstrated that an intervention involving errorless learning during picture naming helped half of the AD patients who received the therapy. In those who benefitted, the effects were sustained for at least 3 months post-treatment. An interesting question is whether over-activity in right posterior perisylvian cortex could have impacted which patients benefited from treatment and, particularly, if decreasing the excitability of this cortex with rTMS or cathodal tDCS could have impacted outcome. While high frequency rTMS in frontal cortex has been used as a treatment for language studies in AD (Cotelli et al., 2006, 2008, 2011), we know of no studies that have applied low frequency rTMS to posterior regions of the right hemisphere to treat language problems in AD.

Finally, cortical inhibition is not the only aging-related process likely to interact with brain injury or disease in older persons. For example, abnormally high amyloid deposition is apparent in approximately 30% of cognitively normal adults over the age of 60, and it is a positively accelerating function of age (Rowe et al., 2010). The area in which such deposition is prominent in cognitively normal adults is in the posterior cingulate/precuneus portion of the default network (e.g., Aizenstein et al., 2008). Sperling et al. (2009) have shown that a lack of a below baseline BOLD response in this region during memory encoding is related to a high level of amyloid deposition. Menke et al. (2009) demonstrated that success of short-term re-acquisition of words in aphasia was positively related to the degree of activity in the left and right hippocampus and parahippocampal gyri, as well as to activity in the right precuneus and posterior cingulate region. The correlation of both posterior cingulate and hippocampal regions with performance is not surprising given the anatomic connections between the two (Parent, 1996). While learning is also correlated with the microstructural integrity of the hippocampus and surrounding white matter (Meinzer et al., 2010), it also would be of interest to determine if posterior cingulate amyloid deposition levels play a role in posterior cingulate and hippocampal activity and in relearning of words in patients with aphasia.

The important points to be made from these three examples, as noted above, are as follows: (1) It is likely that aging × aging-related disease interactions affect more than just motor and language functions. (2) Aging-related processes probably affect patients who have disease and injuries other than stroke. (3) Reduced cortical inhibition is likely not to be the only aging-related process that interacts with injury or disease. In addition to amyloid deposits, for example, deterioration of white matter in aging (e.g., see recent reviews by Bennett and Madden, 2014; Lockhart and DeCarli, 2014; or Nilsson et al., 2014) is a candidate for interacting with aging-related diseases and injury. As discussed below, these points are important for future research regarding the intersection between aging-related processes and rehabilitation.

## Why does it Matter That Aging Interacts with Disease?

In the examples we gave to support the concept that aging-related neural processes interact with brain diseases and injuries in older adults, we developed this argument by citing studies in which low-frequency rTMS was used to downregulate cortical excitability in contralesional cortex. These studies have already shown efficacy as a treatment for language and motor impairments. Based purely on these studies, one might ask: What does it matter if application of low frequency rTMS as a treatment addresses the aging-related component or the disease/injury related component of the disability as long as it is efficacious or effective? The answer is that understanding which component is being targeted is important both for scientific understanding of the phenomenon and for practical reasons in developing new treatments. On the surface, these two reasons may seem to be separable. However, we will develop the argument below that these two facets of the phenomenon are closely related.

Low-frequency rTMS has been shown to decrease cortical excitability (Chen et al., 1997), and high-frequency rTMS has been shown to increase cortical excitability (Wu et al., 2000). On the surface, one might hypothesize that decreasing cortical excitability with low-frequency rTMS should detract from the ability of the target cortical region to participate in tasks that normally might engage it for as long as the effects last, acting as a kind of temporary partial lesion of the area. Conversely, a similar logic suggests that increasing cortical excitability with high-frequency rTMS should increase the ability of a structure to play a role in tasks for the duration of its effects. This simple interpretation has been used as a rationale both for intervention studies and for basic research with rTMS. From a practical standpoint, this reasoning suggests that one could use low-frequency rTMS on a structure when one wishes to reduce the participation of a structure in some specific task and high-frequency rTMS to increase participation of a structure in proximal tasks. This reasoning has even been extended to the long-term effects of rTMS, and similar logic has been used in the application of anodal tDCS, which increases cortical excitability, and cathodal tDCS, which decreases cortical excitability.

However, use only of this logic makes it difficult to interpret some of the findings in the literature. We will use the language literature as a specific example. A growing number of studies indicate that anodal tDCS of (left) Broca’s area/left frontal cortex (e.g., Baker et al., 2010; Marangolo et al., 2013) improves naming of objects or actions more than sham stimulation, either alone or in combination with behavioral aphasia therapy. Anodal tDCS increases cortical excitability, as just noted. However, Monti et al. (2008) found that cathodal tDCS, which decreases cortical excitability, of left frontotemporal structures produces better results on naming than sham stimulation. One candidate explanation for these seemingly contradictory results could be that the location of stimulation between the studies makes a difference. This may in fact be the case, but the interpretation is not as straightforward as it might seem. In many of the studies currently in the literature, the size of the electrodes used in these studies (25–35 cm^2^) makes the electrodes suited to delivering stimulation to a region of cortex as opposed to a specific cortical location. Most investigators have assumed they are stimulating cortical regions directly under these electrodes. However, Datta et al. (2011) showed that because of their relative conductivity, ischemic lesions can change the flow of current through the brain, affecting both perilesional and distant regions. Hence, some of the seemingly contradictory findings might be the product of the way in which the lesion impacts the current flow, and hence which regions are stimulated.^2^ Another possibility is that the assumption that anodal tDCS makes stimulated cortex more likely to participate in tasks spatially and temporally proximal to it and cathodal tDCS makes stimulated cortex less likely to participate in tasks spatially and temporally proximal to it may be too simple.

Further, the mechanisms by which tDCS and rTMS affect remodeling of cortical excitability on a long-term basis have yet to be fully illuminated. However, as noted above, research has indicated a loss of GABA projection neurons and interneurons as a result of aging (Madhusudan et al., 2009; Schmidt et al., 2010; McQuail et al., 2012; Stanley et al., 2012). GABAergic cortical interneurons are ubiquitous. Although much about their effects remains to be understood, it is clear that GABAergic interneurons play an important role in regulating cortical states and processes (Klausberger and Somogyi, 2008). The fact that GABA and GABA_A_ agonists can normalize activity in the visual cortex of older primates (Leventhal et al., 2003) indicates that GABAergic mechanisms play a regulatory role in cortical function that is disturbed by aging. Indeed, GABA seems to be necessary for the selectivity of synaptic changes that occur with learning (Hayama et al., 2013); i.e., a failure to prune non-active dendritic spines during learning would lead to increased nonspecific connectivity, thereby compromising learning. An intriguing possibility is that low-frequency rTMS could help to restore inhibitory tone in contralesional cortex that is lost as a function of aging. The effects of tDCS and why anodal and cathodal tDCS of perilesional cortex both should be an effective treatment in aphasia (see above discussion) require a bit more complex explanation. Anodal stimulation increases glutamate while reducing GABA, and cathodal stimulation appears to reduce both glutamate and GABA levels (Stagg et al., 2009; Clark et al., 2011). Perhaps, the reduction of GABA has a more lasting impact in cathodal tDCS than the reduction of glutamate, leading to a long-term net increase in excitability. Such a mechanism also could explain why both anodal and cathodal tDCS in largely overlapping regions of the damaged hemisphere can have a positive impact on impaired functions; i.e., stimulation may increase long-term excitability, albeit by different mechanisms. Clearly, more research is needed to fully understand how rTMS and tDCS remodel cortical responses during rehabilitation, and how aging interacts with these modalities is critical to understanding their role in aging-related diseases. Hence, it is difficult to understand how we can realize the full potential of rTMS and tDCS as treatment modalities without better understanding their mechanisms of action. Nonetheless, for aging-related diseases, it appears probable that modification of aging-related processes at times plays a role in their effects.

While the above example reveals intriguing possibilities, we again must state that changes in cortical inhibition are not the only aging-related changes in neural processes that could impact rehabilitation. For example, we noted above that amyloid deposition occurs in a large minority of cognitively normal older adults (Aizenstein et al., 2008) and impacts brain activity and behavior (Sperling et al., 2009). Given the fact that stroke is largely a disease of aging adults, it seems highly likely a significant proportion of stroke patients will have amyloid deposition and that the effects of amyloid on cognition will interact with the effects of stroke and impact rehabilitation. This analysis suggests that the level of amyloid deposition in the brains of stroke patients could be predictive of treatment outcome and possibly could impact differentially the effects of various treatments. Clearly, greater understanding of the interaction of this aging-related change following stroke (or other aging-related diseases) would enhance our ability to plan rehabilitation strategies for patients whom it affects. As noted above, white matter deterioration in aging is another candidate for interacting with aging-related diseases. It follows that development of greater knowledge about interactions of other aging-related neural changes with aging-related disease/injury would be useful for rehabilitation research and eventually for treatment of patients as the knowledge bases mature.

## Questions and Strategies for Future Research

We have reviewed data that neurologically normal older persons show the same type of problem suppressing activity in the nondominant hemisphere for language, the nondominant hemisphere for spatial attention, or in M1 ipsilateral to a moving hand that have been hypothesized to be caused by stroke. Such problems that arise following stroke appear to interfere with language, spatial attention, or motor behaviors, and decreasing cortical excitability in the implicated area has therapeutic effects. We suggest that the problems suppressing nondominant-hemisphere cortex for language, spatial attention, or perhaps other cognitive functions or contralesional M1 for movement *may* be due wholly or in part to aging-related changes in the brain and believe that this hypothesis should be the focus of future research studies. Below we discuss implications for future research.

The most obvious implication is that in studies of inhibitory mechanisms for language, neglect, or movement in stroke patients, two types of control groups are necessary: (1) An age-matched control group must be used for comparison to stroke patients to determine if the underlying mechanisms demonstrated by stroke patients are associated with their neurologic insult; (2) A younger control group must be used for comparison to stroke patients and to the age-matched control group to determine if the underlying mechanisms are related to age as opposed to stroke. For example, if we wanted to know if an index of inter-hemispheric inhibition of the unaffected on the affected M1 were age or stroke related, we would need to include the stroke group, an age-matched control group, and a young control group. Use of multiple platforms to measure various aging related phenomena (e.g., loss of inhibitory function, amyloid accumulation, white matter deterioration) also should be considered.

Using both old and young control groups also can be used for assessing brain stimulation as a potential treatment for language, neglect, or motor problems. For example, Wierenga et al. (2008) found that younger and older subjects were equally accurate in picture naming, but that older subjects were slower at naming the pictures. Further, as noted above, they found increased activity in right pars triangularis for older subjects but decreased activity for younger subjects during picture naming. Naeser et al. (2011) looked at suppression of right pars triangularis with low frequency rTMS in nonfluent aphasia patients and in normal controls and found decreased reaction times for picture naming after rTMS in aphasia patients but not in controls. However, their control group was, on the average, more than 20 years younger than their stroke group, confounding age with stroke. Given Wierenga et al. (2008) data, as well as the data of Meinzer et al. (2009, 2012), it is entirely possible that rTMS of right pars triangularis in subjects age-matched to the stroke group would also have shown decreased reaction times with low frequency rTMS of right pars triangularis. Indeed, we are in the process of comparing picture-naming reaction times of younger and older subjects after low frequency rTMS of right pars triangularis. The implications for rehabilitation are that low-frequency rTMS of right pars triangularis during aphasia rehabilitation (or of left inferior parietal cortex for neglect, or of M1 ipsilateral to the moving hand) might work best in older than younger stroke patients because rTMS under these circumstances may be treating the aging side of an aging × aging-related disease interaction. However, this hypothesis will require further research.

Pharmacological methods also could be of use to disentangle underlying mechanisms affecting stroke patients, age-matched controls, and younger subjects. For example, Baclofen and Lorazepam have been used to help determine whether inhibitory phenomena induced by TMS were related to GABA_B_ or GABA_A_ receptor activity, respectively (Di Lazzaro et al., 2004; Irlbacher et al., 2007). Similar studies using a broader range of agents could be used to develop knowledge about inhibitory mechanisms in aging-related diseases, such as stroke.

We have focused much attention in this review on NIBS (i.e., rTMS, tDCS) because the close parallels with recent aging research raise important questions regarding the role of aging in rehabilitation of aging related diseases. However, it is appropriate to consider other means of affecting the aging component in rehabilitation of aging-related disease. For example, aerobic exercise and the resultant levels of increased cardiovascular fitness have proven to be one of the most robust interventions to improve cognition and enhance related brain activity in older adults, and it also appears to have protective effects against developing neurodegenerative diseases (Kramer and Erickson, 2007; Erickson and Kramer, 2009). As noted, our cross-sectional studies indicate that exercise may mitigate changes in inhibitory mechanisms in older adults (McGregor et al., 2011, 2012, 2013). We have also demonstrated increased semantic fluency output following an aerobic exercise intervention in previously sedentary older adults (Nocera et al., 2015). This finding is important in the context that we have shown sedentary older adults displayed reductions in negative task-related activity in areas of the attention network when performing an overt semantic fluency task during fMRI. Further, in these same sedentary participants, longer interhemispheric inhibition was associated with more negative task-related activity in the right and left posterior perisylvian cortex, suggesting that sedentary aging may result in losses in task facilitatory cortical inhibition during language tasks (Zlatar et al., 2013). Importantly, and in line with our previous work, these losses were mitigated in older adults with higher levels of cardiovascular fitness. A longitudinal study is ongoing to determine if aerobic exercise can be used as an intervention to restore inhibitory mechanisms in older adults and improve the neural substrates of language. If aerobic exercise is an effective intervention in this regard, it would offer an intervention for reversing loss of inhibitory mechanisms during rehabilitation for aging-related diseases.

Finally, one might consider whether behavioral treatments could be devised to address the aging component in rehabilitation of aging-related disease. Crosson et al. (2007) developed an aphasia treatment to re-lateralize activity from the left to the right frontal lobe by pairing complex left-hand movements with word-finding trials during treatment. This “intention” treatment improved word finding (Crosson et al., 2007; Benjamin et al., 2014) and did shift lateral frontal laterality rightward (Crosson et al., 2009; Benjamin et al., 2014). These findings may seem contrary to analyses earlier in this review. However, almost all of the right frontal activity post-treatment was located in M1, premotor cortex, or pars opercularis (Crosson et al., 2009), which is posterior to pars triangularis, the target for reducing cortical excitability in rTMS aphasia treatment studies (Naeser et al., 2005; Barwood et al., 2011; Thiel et al., 2013). Further, right frontal activity was often reduced even in patients for whom laterality indices shifted rightward or for whom frontal activity was confined to the right hemisphere both pre- and post-treatment. These latter findings suggest an increased focus of activity in posterior portions of the right frontal lobe. Naeser et al. (2011) found that decreasing right frontal excitability with low-frequency rTMS posterior to right pars triangularis in patients with aphasia hampered (slowed) picture naming. Hence, when the localization of right frontal activity post-treatment is considered, findings from this “intention” treatment are consistent with the rTMS literature in aphasia. More to the point of this discussion is the idea that a behavioral treatment might be invoked to target specific goals with respect to remodeling cortical activity. A good deal more research is necessary with this “intention” treatment to verify its effects, and there certainly are many more behavioral strategies that could be used to induce plasticity in specific cortical targets. In other words, use of behavioral techniques to target aging-related changes during rehabilitation is a potentially productive area for future research.

### Conclusions

In our final remarks, we will sharpen the focus of our conclusions. To avoid misunderstanding, we must clarify what we are implying, but also make clear the boundaries and limitations to those implications. First, our intent is to introduce the possibility that in some cases, rTMS and/or tDCS can be used to lower the impact of aging-related processes on disease-/injury-induced cognitive and motor impairments in older adults. Specifically, in stroke and perhaps other aging-related diseases, reduction of excitability in the non-dominant hemisphere for the function in question may work because it is addressing an aging-related change that compounds the lesion or other effect of the disease/injury. A potential explanation for this phenomenon is that aging-related activity in the target non-dominant cortex introduces noise into a system where the signal-to-noise ratio is compromised by the disease/injury. However, a caveat is that increasing the excitability of perilesional cortex through rTMS or tDCS is probably treating the disease/injury-related side of the equation, perhaps by enhancing signal in the system. Also, implicit in our comments is the idea that decreasing nondominant-hemisphere activity in normal older adults with rTMS or tDCS could be used to increase their efficiency in cognitive and motor performance, although the effects would be smaller because the synergistic deficits of the aging-related brain disease are not present. Second, we offer our inferences as hypotheses rather than as proven fact. These hypotheses will have the greatest impact on the field of neuro-rehabilitation if they are adequately tested and affirmed. In other words, we believe that a healthy debate about the propositions we have set forward could help to focus rehabilitation research for aging-related diseases in the future. Third, we see addressing aging-related phenomena in rehabilitation as another potential tool to lessen the impact of aging-related disease/injury on our patients. We should avoid seeing aging-related phenomena as a panacea for rehabilitation of aging-related disease or injury in older adults. Nonetheless, research into this area has the potential for considerable impact on neuro-rehabilitation. Fourth, we have used existing rehabilitation research with rTMS and tDCS to illustrate that addressing the aging-related component of an aging-related by disease/injury interaction may advance rehabilitation research and treatment. However, there probably are many other modalities through which aging-related effects can be addressed. For example, we have discussed aerobic exercise as another method for mitigating the aging-related component. It also is worth pointing out that we have cited studies where traditional therapies to address the disease-related component of the deficit along with rTMS or tDCS are emerging in the literature (e.g., Conforto et al., 2012; Etoh et al., 2013; Thiel et al., 2013; Barros Galvão et al., 2014). Fifth, we have used examples from research in stroke and AD to illustrate how aging-related processes may impact cognitive and motor impairments and the potential impact of treating the aging-related component of the impairments. However, there are likely numerous other aging-related diseases to which the principles covered in this paper can be applied. Sixth, when proof of concept papers for rehabilitation of aging-related diseases are done on neurologically normal participants, older adults are the appropriate group, though younger adults may be needed to assess the impact of aging on the phenomenon under study.

Finally, one of the main themes of this review is that further investigation of the interaction between aging-related processes and disease/injury processes in rehabilitation is needed. One problem in advancing this agenda is that cross-fertilization is infrequent for aging research and research on rehabilitation of aging-related diseases. Specifically, distinguishing the effects of aging from the effects of aging-related disease/injury is rarely a design consideration in rehabilitation research. A potential way of overcoming this problem would be to develop conferences designed to increase the cross-fertilization between aging investigators and rehabilitation investigators. Figures from the US Census Bureau indicate that in 2010, almost 57 million Americans (18.4% of the population) were 60 years of age or older. By 2050, the number of Americans of this age will nearly double to approximately 112 million (25.5% of the population; Administration on Aging, 2010). As we continue to develop medical treatments to increase the survivability of stroke, slow the progress of AD, mitigate the effects of PD, and lessen the impact of other aging-related diseases and injuries, rehabilitation for these problems will become increasingly important to preserve quality of life and reduce the economic and social impact of aging-related diseases and injuries. We believe that addressing aging-related processes in the rehabilitation of these and other aging-related disorders eventually can yield powerful tools to assist in accomplishing this goal.

## Conflict of Interest Statement

The authors declare that the research was conducted in the absence of any commercial or financial relationships that could be construed as a potential conflict of interest.

## Footnotes

^1^It is of interest that Rosen et al. (2000) based this conclusion on comparison of their aphasic patients with neurologically normal controls. However, it must be noted that their two control groups were an average of 11 and 23 years younger than their patients. The inadvisability of using *only* younger subjects as controls for stroke patients will become obvious shortly. Indeed, this point is central to the main theme of this brief review.

^2^It is worth noting that Dmochowski et al. (2013) have developed current flow models for delivering tDCS to target cortical regions in stroke patients. The models involve use of newer tDCS methods involving multiple smaller electrodes that make it easier to target specific cortical areas. As more studies accumulate using these methods, greater clarity and consistency of effects may emerge from the tDCS literature.
